# Risk factors for low cardiac output syndrome in children with congenital heart disease undergoing cardiac surgery: a retrospective cohort study

**DOI:** 10.1186/s12887-020-1972-y

**Published:** 2020-02-24

**Authors:** Xinwei Du, Hao Chen, Xiaoqi Song, Shunmin Wang, Zedong Hao, Lifeng Yin, Zhaohui Lu

**Affiliations:** 1 Department of Cardiothoracic Surgery, Shanghai Children’s Medical Center, School of Medicine, Shanghai Jiao Tong University, 1678 Dongfang Road, Pudong district, Shanghai, China; 2 Synyi Medical Technology, Shanghai, China

**Keywords:** Low cardiac output syndrome, Congenital heart disease, Risk factor, Cardiac surgery, Left ventricular ejection fraction, Cardiopulmonary bypass

## Abstract

**Background:**

Low cardiac output syndrome (LCOS) is an important complication of cardiac surgery. It is associated with increased morbidity and mortality. The incidence of LCOS after surgery is high in patients with congenital heart disease (CHD). Therefore, determining the risk factors of LCOS has clinical significance for the management of CHD. This study aimed to analyze the risk factors of LCOS.

**Methods:**

We conducted a retrospective analysis of children with CHD who underwent cardiac surgery at Shanghai Children’s Medical Center between January 1, 2014, and December 31, 2017. Demographic characteristics and baseline data were extracted from the health data resource center of the hospital, which integrates clinical routine data including medical records, diagnoses, orders, surgeries, laboratory tests, imaging, nursing, and other subsystems. Logistic regressions were performed to analyze the risk factors of LCOS.

**Results:**

Overall, 8660 infants with CHD were included, and 864 (9.98%) had LCOS after surgery. The multivariate regression analysis identified that age (OR 0.992, 95% CI: 0.988–0.997, *p* = 0.001), tricuspid regurgitation (1.192, 1.072–1.326, *p* = 0.001), Risk Adjustment in Congenital Heart Surgery-1 risk grade (1.166, 1.011–1.345, *p* = 0.035), aortic shunt (left-to-right: 1.37, 1.005–1.867, *p* = 0.046; bi-directional: 1.716, 1.138–2.587, *p* = 0.01), atrial shunt (left-to-right: 1.407, 1.097–1.805, *p* = 0.007; right-to-left: 3.168, 1.944–5.163, *p* < 0.001; bi-directional: 1.87, 1.389–2.519, *p* < 0.001), ventricular level shunt (left-to-right: 0.676, 0.486–0.94, *p* = 0.02; bi-directional: 2.09, 1.611–2.712, *p* < 0.001), residual shunt (3.489, 1.502–8.105, *p* = 0.004), left ventricular outflow tract obstruction (3.934, 1.673–9.254, *p* = 0.002), right ventricular outflow tract obstruction (3.638, 1.225–10.798, *p* = 0.02), circulating temperature (mild hypothermia: 1.526, 95% CI: 1.205–1.934, *p* < 0.001; middle and low temperature: 1.738, 1.236–2.443, *p* = 0.001), duration of cardiopulmonary bypass (1.009, 1.006–1.012, *p* < 0.001), myocardial preservation using histidine-tryptophan-ketoglutarate (1.677, 1.298–2.167, *p* < 0.001), and mitral insufficiency (1.714, 1.239–2.37, *p* < 0.001) were independent risk predictors of LCOS.

**Conclusions:**

The incidence of postoperative LCOS in CHD children remains high. Circulation temperature, myocardial preservation using histidine-tryptophan-ketoglutarate, and usage of residual shunt after surgery were independent risk predictors for LCOS.

## Background

Low cardiac output syndrome (LCOS) is the most common complication following cardiac surgeries. The incidence of LCOS in the surgical treatment of congenital heart disease (CHD) has been reported to be 25–60% [1], associated with a significant risk of mortality among patients [2, 3]. LCOS is associated with a decreased ejection fraction and decreased oxygen supply, which may cause hypoxia. The patients are often at a high risk of mortality and require more intensive care such as extended stay in the intensive care unit (ICU) and ventilatory support.

The etiology of postoperative LCOS is multifactorial [4]. Pathologically, endothelial dysfunction and myocardial stunning, acute changes in the loading conditions of the myocardium, use of cardioplegia, activation of the inflammatory and complement cascade caused by cardiopulmonary bypass (CPB), and the residual hemodynamic burden of uncorrected defects [5] have been suggested to be the causes of LCOS. The most commonly reported clinical predictors of LCOS include left ventricular ejection fraction < 20%, surgical history, female gender, and increasing age [2, 6, 7]. Prediction models based on clinical data, such as EuroSCORE, have been proposed [8]. However, the risk factors of LCOS are varied in the literature according to the population and surgery categories included [2, 9–11].

To date, only a few studies presented the detailed analysis of the risk factors for postoperative LCOS and the further complications in children with CHD. This study aimed to analyze the risk factors for LCOS and to determine their association with the incidence of postoperative death, extended ICU stay, and the duration of mechanical ventilator support in Chinese children with and without LCOS.

## Methods

### Study design and population

In this retrospective study, patients with CHD who underwent cardiac surgery at Shanghai Children’s Medical Center from January 1, 2014, to December 31, 2017, were included. Patients aged ≥18 years, on CPB, who had undergone general thoracic surgery not involving cardiotomy, or with uncertain in-hospital survival records were excluded from the study. The study was approved by the Ethical Committee of Shanghai Children’s Medical Center. Demographic characteristics and baseline data were extracted from the health data resource center of the hospital, which integrated clinical routine data including medical records, diagnoses, orders, operations, laboratory tests, imaging, nursing, and other subsystems.

Patients were classified into two groups, based on whether they developed LCOS into the LCOS and non-LCOS groups. The baseline characteristics and intraoperative features of the two groups were compared. Associations of these characteristics with LCOS were analyzed using regression analysis. In addition, the mortality, extended ICU stay, and duration of mechanical ventilator support were compared between the two groups.

### Outcomes

LCOS is characterized by clinical signs or symptoms including elevated blood lactate or rapid increase in blood lactate, decreased central venous oxygen saturation, increased arterial to central venous oxygen saturation difference, decreased urine output, increased peripheral skin temperature to core body temperature difference, and low echocardiographic Doppler-derived cardiac index, high inotrope requirement [12]. In-hospital mortality was defined as death during the same hospitalization regardless of cause. Prolonged ICU stay was defined as > 3 days of ICU stay, and extended duration of mechanical ventilation support was defined as > 48 h’ ventilatory support [13].

### Risk factors

The risk factors of LCOS analyzed in this study include baseline and demographic data, pre-operative Doppler echocardiography characteristics, characteristics of surgery and CPB, as well as postoperative measures of CHD. The body mass index (BMI), as categorical data percentiles varied according to age groups, which were age-adjusted according to the pediatric BMI reference data for China [14]. Moreover, the Risk Adjustment in Congenital Heart Surgery-1 (RACHS-1) score was defined as ordinal data; an increase in the RACHS-1 score can indicate the risk of LCOS [15]. Nutrition status has been recognized as an important risk factor of postoperative complications in CHD surgery. In this study, the incidences of LCOS in CHD children with different nutrition status were analyzed separately.

### Statistical analysis

Continuous variables are expressed as mean ± standard deviation (SD) (normally distributed) or median (IQR) (non-normally distributed). Categorical variables are presented as frequency (%). Depending on the distributions, the t-test or Mann-Whitney U test was used to compare the continuous variables between the groups. In addition, a chi-squared test or Fisher’s exact test was used for the comparison of categorical variables.

Univariate logistic regressions with LCOS as an outcome were analyzed first. Then, the Variance Inflation Factor was calculated to explore the independence of the selected variables. The results are listed in Supplemental Table 1, and there is no evidence to show dependence among the selected factors. Therefore, the significant variables were entered into multiple logistic regressions without an interaction term, and the stepwise variable selection method was used to identify the potential risk factors of LCOS. All tests were two-sided, and *P* < 0.05 was considered as statistically significant. All analyses were performed using SAS software, version 9.4 (SAS Institute INC).

## Results

### Demographic characteristics of CHD children

Overall, 8660 children were included in the study, with 864 (9.98%) LCOS cases after surgery. The demographic characteristics at baseline including age (*p* < 0.001) and body mass index (BMI) <5th percentile (*p* = 0.016) were significantly different between the CHD children with LCOS and those without LCOS (Table 1).

**Table 1:** The clinical characteristics of CHD children (including demographic characteristics, pre-operative color doppler echocardiography characteristics, the characteristic of surgery and cardiopulmonary bypass, and post-operative characteristics)

Parameter		LCOS (*n* = 864)	non-LCOS (*n* = 7796)	Total (*n* = 8660)	*P* value
*Demographic characteristics*					
Age (days)	Median (IQR)	206.5 (94,507.5)	350 (182, 950.5)	329 (173, 916)	< 0.001
Gender	MALE	56.4%	55.0%	55.1%	0.4
	FEMALE	43.6%	45.1%	44.9%	
BMI	<5th percentile	23.4%	19.0%	19.5%	0.016
	5th~95th percentile	62.8%	66.7%	66.3%	
	>95th percentile	13.8%	14.3%	14.2%	
*Pre-operative color doppler echocardiography characteristics*					
Aortic shunt	None	76.1%	90.4%	89.0%	< 0.001
	Left-to-right	15.3%	7.7%	8.4%	
	Right-to-left	0.7%	0.03%	0.1%	
	Bi-directional	8.0%	1.9%	2.5%	
Atrial shunt	None	21.7%	36.5%	35.1%	< 0.001
	Left-to-right	39.6%	47.8%	47.0%	
	Right-to-left	6.9%	2.7%	3.1%	
	Bi-directional	31.8%	13.0%	14.9%	
Ventricular level shunt	None	23.7%	28.6%	28.1%	< 0.001
	Left-to-right	12.3%	40%	37.3%	
	Right-to-left	0.3%	0.1%	0.1%	
	Bi-directional	63.8%	31.3%	34.5%	
AI	Negative	80.0%	83.1%	82.8%	0.035
	Minimal	13.6%	10.8%	11.0%	
	Mild	5.2%	5.2%	5.2%	
	Mild-to-moderate	0.3%	0.5%	0.4%	
	Moderate	0.4%	0.3%	0.3%	
	Moderate-to-severe	0.1%	0.1%	0.1%	
	Severe	0.5%	0.1%	0.1%	
MR	Negative	26.6%	22.6%	23.0%	0.9
	Minimal	43.0%	49.0%	48.4%	
	Mild	18.5%	20.5%	20.3%	
	Mild-to-moderate	6.1%	4.5%	4.6%	
	Moderate	3.3%	2.3%	2.4%	
	Moderate-to-severe	1.7%	0.7%	0.8%	
	Severe	0.8%	0.4%	0.4%	
PI	Negative	16.3%	9.2%	9.9%	0.007
	Minimal	53.1%	59.5%	58.9%	
	Mild	28.8%	29.6%	29.6%	
	Mild-to-moderate	0.7%	0.6%	0.6%	
	Moderate	1.1%	1.0%	1.0%	
	Moderate-to-severe	0%	0.03%	0.03%	
	Severe	0%	0.04%	0.04%	
TR	Negative	1.2%	0.4%	0.5%	0.001
	Minimal	42.8%	47.4%	46.9%	
	Mild	40.3%	43.3%	43%	
	Mild-to-moderate	8.5%	5.6%	5.8%	
	Moderate	4.4%	2.4%	2.6%	
	Moderate-to-severe	2.0%	0.4%	0.6%	
	Severe	0.8%	0.5%	0.6%	
LVEF	< cut off value	56.3%	55.4%	55.5%	0.7
	Normal	27.3%	28.7%	28.5%	
	> cut off value	16.5%	15.9%	16%	
LVFS	< cut off value	56.9%	54.2%	54.4%	0.1
	Normal	27.5%	30.9%	30.6%	
	> cut off value	15.6%	14.9%	15.0%	
Mitral insufficiency	NO	92.0%	93.8%	93.6%	0.044
	YES	8.0%	6.2%	6.4%	
Mitral Stenosis	NO	99.0%	99.4%	99.4%	0.1
	YES	1.0%	0.6%	0.6%	
*The characteristic of surgery and cardiopulmonary bypass*					
History of heart surgery	NO	84.6%	92.3%	91.5%	< 0.001
	YES	15.4%	7.7%	8.5%	
RACHS-1 risk grade	1	6.0%	13.9%	13.1%	< 0.001
	2	50.1%	62.8%	61.6%	
	3	32.4%	20.9%	22.1%	
	4	10.3%	2.2%	3.0%	
	5	1.2%	0.1%	0.2%	
	6	0%	0.1%	0.1%	
CPB duration (min)	Median (IQR)	87 (62,119)	51 (38,72)	53 (39,78)	< 0.001
Aortic clamping time (min)	Median (IQR)	53 (37,74)	29 (20,44)	31 (21,48)	< 0.001
Operation characteristics	Selective extracorporeal surgery	92.8%	98.6%	98%	< 0.001
	Emergency non-extracorporeal surgery	0.1%	0.01%	0.02%	
	Emergency extracorporeal surgery	7.1%	1.2%	1.8%	
	Selective non-extracorporeal surgery	0%	0.1%	0.1%	
	Elective thoracotomy	0%	0.1%	0.1%	
Circulating temperature	Normal temperature	21.8%	53.1%	49.9%	< 0.001
	Mild hypothermia	52.0%	38.7%	40.1%	
	Deep hypothermia	2.2%	0.6%	0.7%	
	Middle and low temperature	24.1%	7.6%	9.3%	
Circulation method	Low flow cycle	0.7%	0.1%	0.1%	< 0.001
	Cerebral perfusion	5.3%	1.5%	1.9%	
	Parallel CPB	3.0%	3.4%	3.3%	
	Full flow	89.9%	94.6%	94.2%	
	Circulatory arrest	0.9%	0.4%	0.4%	
	Cerebrovascular perfusion	0.1%	0.1%	0.1%	
Difficult to wean from CPB	NO	99.7%	99.9%	99.9%	0.1
	YES	0.4%	0.1%	0.1%	
Myocardial preservation	HTK	31.6%	8.9%	11.2%	< 0.001
	Cold blood cardioplegia (4:1)	68.4%	91.1%	88.8%	
*Post-operative characteristic*					
Residual shunt	NO	98.4%	99.6%	99.5%	< 0.001
	YES	1.62%)	0.4%	0.5%	
LVOTO	NO	98.3%	99.5%	99.4%	< 0.001
	YES	1.7%	0.5%	0.6%	
RVOTO	NO	98.4%	99.7%	99.6%	< 0.001
	YES	1.6%	0.3%	0.4%	
Postoperative rhythm of the heart	III°AVB	1.2%	0.1%	0.2%	< 0.001
	II°AVB	0.6%	0.2%	0.2%	
	Atrioventricular dissociation	0.2%	0.1%	0.1%	
	Supraventricular tachycardia	0.2%	0.1%	0.1%	
	Sinus rhythm	97.8%	99.5%	99.3%	

*Abbreviation*: *CHD* Congenital Heart Disease, *LCOS* Low Cardiac Output Syndrome, *BMI* Body Mass Index, *AI* Aortic Insufficiency, *MR* Mitral Regurgitation, *PI* Pulmonary Insufficiency, *TR* Tricuspid Regurgitation, *LVEF* Left Ventricular Ejection Fraction, *LVFS* left ventricular fraction shortening, *CPB* Cardiopulmonary Bypass, *LVOTO* Left Ventricular Outflow Tract Obstruction, *RVOTO* Right Ventricular Outflow Tract Obstruction, *HTK* histidine-tryptophan-ketoglutarate, *AVB* atrioventricular block

### Preoperative Doppler echocardiography characteristics of CHD children

When Doppler echocardiography characteristics were compared between the CHD children in the two groups, it was found that aortic shunt (*p* < 0.001), atrial shunt (*p* < 0.001), ventricular level shunt (*p* < 0.001), aortic insufficiency (*p* = 0.035), pulmonary insufficiency (*p* = 0.007), tricuspid regurgitation (TR) (*p* = 0.001), and mitral insufficiency (*p* = 0.044) of LCOS patients were significantly different from those of non-LCOS patients (Table 1).

### Characteristics of surgery and cardiopulmonary bypass of CHD children

In LCOS patients, operation characteristics (*p* < 0.001), procedure complexity (represented by RACHS-1 risk grade) (*p* < 0.001), CPB duration (*p* < 0.001), aortic clamping time (< 0.001), history of heart surgery (*p* < 0.001), circulating temperature (*p* < 0.001), circulation method (< 0.001), and myocardial preservation (*p* < 0.001) were significantly different from those without LCOS (Table 1).

### Postoperative measures of CHD children

There were significant differences in postoperative residual shunt (*p* < 0.001), left ventricular outflow tract obstruction (LVOTO, *p* < 0.001), right ventricular outflow tract obstruction (RVOTO, *p* < 0.001), and postoperative rhythm of the heart (*p* < 0.001) between LCOS and non-LCOS patients (Table 1).

### Logistic regression analysis

Univariate analysis identified that age, aortic insufficiency, pulmonary insufficiency, TR, RACHS-1 risk grade, aortic shunt, atrial shunt, ventricular level shunt, emergency extracorporeal surgery, history of heart surgery, residual shunt, LVOTO, RVOTO, abnormal circulation temperature, circulation method (except for parallel CPB and cardio-cerebral perfusion), duration of CPB, aortic clamping time, postoperative rhythm of the heart (III°AVB, II°AVB), difficult to wean from CPB, myocardial preservation using histidine-tryptophan-ketoglutarate (HTK), BMI <5th percentile, and mitral insufficiency were significantly associated with LCOS in CHD children (Table 2).

**Table 2:** Univariate/multivariate logistic regression analysis for the association of pre−/post-operative characteristics with LCOS

		Univariate analysis		Multivariate analysis	
Parameter		OR (95% CI)	*P*-value	OR (95% CI)	*P*value
Age (days)		0.992 (0.989,0.995)	< 0.001	0.992 (0.988,0.997)	0.001
Gender	Male vs Female	1.06 (0.92,1.22)	0.4		
AI		1.118 (1.008,1.239)	0.034		
MR		1.06 (0.99,1.14)	0.1		
PI		0.845 (0.751,0.951)	0.005		
TR		1.226 (1.13,1.33)	< 0.001	1.192 (1.072,1.326)	0.001
RACHS-1 risk grade		2.094 (1.903,2.304)	< 0.001	1.166 (1.011,1.345)	0.035
Aortic shunt	Left-to-right vs None	2.363 (1.904,2.932)	< 0.001	1.37 (1.005,1.867)	0.046
	Right-to-left vs None	27.469 (5.318,141.869)	< 0.001		
	Bi-directional vs None	5.087 (3.721,6.953)	< 0.001	1.716 (1.138,2.587)	0.01
Atrial shunt	Left-to-right vs None	1.396 (1.151,1.693)	0.001	1.407 (1.097,1.805)	0.007
	Right-to-left vs None	4.412 (3.149,6.18)	< 0.001	3.168 (1.944,5.163)	< 0.001
	Bi-directional vs None	4.111 (3.34,5.059)	< 0.001	1.87 (1.389,2.519)	< 0.001
Ventricular level shunt	Left-to-right vs None	0.37 (0.288,0.476)	< 0.001	0.676 (0.486,0.94)	0.02
	Right-to-left vs None	3.16 (0.65,15.32)	0.2	3.458 (0.452,26.448)	0.2
	Bi-directional vs None	2.452 (2.052,2.93)	< 0.001	2.09 (1.611,2.712)	< 0.001
Operation characteristics	Emergency non-extracorporeal surgery vs Selective extracorporeal surgery	9.58 (0.6153.34)	0.1		
	Emergency extracorporeal surgery vs Selective extracorporeal surgery	6.153 (4.425,8.556)	< 0.001		
	Selective non-extracorporeal surgery vs Selective extracorporeal surgery	0 (0,1.053157E255)	0.97		
	Elective thoracotomy vs Selective extracorporeal surgery	0 (0)	0.98		
History of heart surgery	YES vs NO	2.17 (1.772,2.658)	< 0.001		
Residual shunt	YES vs NO	4.264 (2.252,8.073)	< 0.001	3.489 (1.502,8.105)	0.004
LVOTO	YES vs NO	3.81 (2.078,6.988)	< 0.001	3.934 (1.673,9.254)	0.002
RVOTO	YES vs NO	5.82 (2.967,11.418)	< 0.001	3.638 (1.225,10.798)	0.02
Circulating temperature	Mild hypothermia vs Normal temperature	3.271 (2.741,3.903)	< 0.001	1.526 (1.205,1.934)	< 0.001
	Deep hypothermia vs Normal temperature	9.289 (5.328,16.194)	< 0.001	1.369 (0.587,3.194)	0.467
	Middle and low temperature vs Normal temperature	7.691 (6.202,9.537)	< 0.001	1.738 (1.236,2.443)	0.001
Circulation method	Low flow vs Full flow	11.395 (3.47,37.422)	< 0.001		
	Cerebral perfusion vs Full flow	3.671 (2.591,5.199)	< 0.001		
	Parallel CPB vs Full flow	0.94 (0.63,1.42)	0.8		
	Circulatory arrest vs Full flow	2.813 (1.274,6.214)	0.011		
	Cardio-cerebral Perfusion vs Full flow	1.90 (0.22,16.28)	0.6		
CPB duration (min)		1.016 (1.014,1.017)	< 0.001	1.009 (1.006,1.012)	< 0.001
Aortic clamping time (min)		1.026 (1.023,1.028)	< 0.001		
Postoperative rhythm of the heart	III°AVB vs Sinus rhythm	9.181 (3.81,22.121)	< 0.001		
	II°AVB vs Sinus rhythm	2.869 (1.048,7.851)	0.04		
	Atrioventricular dissociation vs Sinus rhythm	2.30 (0.49,10.83)	0.3		
	Supraventricular tachycardia vs Sinus rhythm	4.59 (0.84,25.1)	0.1		
Difficult to wean from CPB	YES vs NO	4.524 (1.129,18.121)	0.033		
Myocardial preservation	HTK vs Cold blood cardioplegia (4:1)	4.744 (4.014,5.607)	< 0.001	1.677 (1.298,2.167)	< 0.001
BMI	<5th percentile vs 5th~5th percentile	1.305 (1.086,1.569)	0.004		
	>95th percentile vs 5th~5th percentile	1.03 (0.82,1.28)	0.8		
LVEF	< cut off value vs Normal	1.07 (0.90,1.27)	0.4		
	> cut off value vs Normal	1.09 (0.87,1.37)	0.5		
LVFS	< cut off value vs Normal	1.18 (1.00,1.4)	0.1		
	> cut off value vs Normal	1.18 (0.93,1.48)	0.2		
Mitral insufficiency	YES vs NO	1.309 (1.006,1.702)	0.045	1.714 (1.239,2.37)	0.001
Mitral stenosis	YES vs NO	1.77 (0.87,3.64)	0.1		

*Abbreviation*: *OR* Odds Ratio, *CI* Confidence Interval, *LCOS* Low Cardiac Output Syndrome, *BMI* Body Mass Index, *AI* Aortic Insufficiency, *MR* Mitral Regurgitation, *PI* Pulmonary Insufficiency, *TR* Tricuspid Regurgitation, *LVEF* Left Ventricular Ejection Fraction, *LVFS* left ventricular fraction shortening, *CPB* Cardiopulmonary Bypass, *LVOTO* Left Ventricular Outflow Tract Obstruction, *RVOTO* Right Ventricular Outflow Tract Obstruction, *HTK* histidine-tryptophan-ketoglutarate, *AVB* atrioventricular block

Multivariate analysis of these variables found age (OR 0.992, 95% CI: 0.988–0.997, *p* = 0.001), TR (OR 1.192, 95% CI 1.072–1.326, *p* = 0.001), RACHS-1 risk grade (OR 1.166, 95% CI 1.011–1.345, *p* = 0.035), aortic shunt (left-to-right: OR 1.37, 95% CI 1.005–1.867, *p* = 0.046; bi-directional: OR 1.716, 95% CI 1.138–2.587, *p* = 0.01), atrial shunt (left-to-right: OR 1.407, 95% CI 1.097–1.805, *p* = 0.007; right-to-left: OR 3.168, 95% CI 1.944–5.163, *p* < 0.001; bi-directional: OR 1.87, 95% CI 1.389–2.519, *p* < 0.001), ventricular level shunt (left-to-right: OR 0.676, 95% CI 0.486–0.94, *p* = 0.02; bi-directional: OR 2.09, 95% CI 1.611–2.712, *p* < 0.001), residual shunt (OR 3.489, 95% CI 1.502–8.105, *p* = 0.004), LVOTO (OR 3.934, 95% CI 1.673–9.254, *p* = 0.002), RVOTO (OR 3.638, 95% CI 1.225–10.798, *p* = 0.02), circulating temperature (mild hypothermia: OR 1.526, 95% CI 1.205–1.934, *p* < 0.001; middle and low temperature: OR 1. 738, 95% CI 1.236–2.443, *p* = 0.001), duration of CPB (OR 1.009, 95% CI 1.006–1.012, *p* < 0.001), myocardial preservation using HTK (OR 1.677, 95% CI 1.298–2.167, *p* < 0.001), and mitral insufficiency (OR 1.714, 95% CI 1.239–2.37, *p* < 0.001) were independent risk predictors of LCOS (Table 2).

### Postoperative mortality, prolonged ICU stay, and extended medical ventilator support in LCOS and non-LCOS CHD children

The mortality rate (7.18% vs. 1.08%, *p* < 0.001), ICU stay > 3 days (89.24% vs. 33.35%, *p* < 0.001), and duration of mechanical ventilator support > 48 h (93.21% vs. 44.5%, *p* < 0.001) of LCOS children were significantly higher than those of non-LCOS children (Table 3).

**Table 3:** The post-operative mortality, length of ICU > 3d, duration of Medical ventilator > 48 h in LCOS and non-LCOS CHD children

Parameter		LCOS (*n* = 864)	Non- LCOS (*n* = 7796)	Total (*n* = 8660)	*P* value
Mortality (%)	NO	92.8%	98.9%	98.3%	< 0.001
	YES	7.2%	1.1%	1.7%	
Length of ICU stay	<=3d	10.8%	66.7%	61.1%	< 0.001
	> 3d	89.2%	33.4%	38.9%	
Duration of Medical ventilator	<=48 h	6.8%	55.5%	50.7%	< 0.001
	> 48 h	93.2%	44.5%	49.3%	

*Abbreviation*: *ICU* Intensive Care Unit, *LCOS* Low Cardiac Output Syndrome, *CHD* Congenital Heart Disease

### Incidence of postoperative LCOS in children with CHD of different nutritional status

In CHD children with BMI <5th percentile, the incidences of LCOS showed a gradually decreasing trend in neonates aged > 3 months. In neonates aged 3–6 months, the incidence increased, declined in children aged 6 months to > 3 years old. In patients with BMI 5th~95th percentile and > 95th percentile, the incidences of LCOS showed a gradually decreasing trend. (Table 4).

**Table 4:** Incidence of postoperative LCOS in children with CHD of different nutritional status

Age	BMI < 5th percentile	BMI 5th~95th percentile	BMI > 95th percentile
< 1 month	41.2%	39.4%	35.7%
1 month~ 3 month	17.9%	26.1%	17.9%
3 month~ 6 month	21.4%	12.5%	9.1%
6 month~ 1 year	11.5%	9.3%	5.1%
1 year~ 3 year	10.2%	4.4%	4.2%
> 3 year	12.6%	7.8%	3.1%

*Abbreviation*: *3.LCOS* Low Cardiac Output Syndrome, *CHD* Congenital Heart Disease

## Discussion

In our study, the incidence of LCOS was 9.98%, which is higher than that in adult patients postoperatively (2.4–9.1%) [2, 3, 7], but lower than that of another study on newborns, in which the LCOS incidence at 36 h after surgery was 25.9, 17.5, and 11.7% of infants (median age, 3 months) on placebo, low-dose milrinone, and high-dose milrinone, respectively [16]. In a secondary retrospective analysis of a prospective randomized trial, LCOS occurred in 32 of 76 (42%) neonates (median age, 7 days) after cardiac surgery [17]. This difference was attributed to the probable difference in age. Neonatal myocardium is physiologically immature [18–21] and every procedure affects the incidence of LCOS differently [22]. The age range of children included in this study was larger (1–6457 days), and we included different types of cardiac surgery. This could be the main reason why the LCOS incidence was higher than that in adults and lower than that in newborns.

We found that age, aortic shunt (left-to-right and bi-directional), atrial shunt, ventricular level shunt (left-to-right and bi-directional), circulation temperature, and myocardial preservation using HTK were independent predictors of LCOS.

The risk for LCOS was higher for neonates given that their myocardial development is not complete. The effect of age on the risk of LCOS in adults has been confirmed earlier. In a prospective study, multivariate analysis showed that age was an independent predictor of LCOS [9] and a retrospective study ascertained that increasing age is an independent predictor of LCOS [2].

By preoperative Doppler echocardiography characteristics of CHD children, aortic shunt (left-to-right and bi-directional), atrial shunt, ventricular level shunt (left-to-right and bi-directional) were deduced as significant risk factors for LCOS. Left-to-right shunt before surgery may result in right heart failure, leading to low cardiac output. Xiong et al. found that preoperative right-left shunt ventricular septal defect was a risk factor of LCOS [23]. Ischemic mitral valve pathology was found as an independent predictor for LCOS after isolated mitral valve surgery [3].

We also found that the circulation temperature (mild hypothermia, middle and low temperature) and the proportion of myocardium preserved using HTK were independent predictors of LCOS. During routine hypothermic CPB, the heart is damaged by hypothermia, ischemia-reperfusion, or hyperkalemia, which is associated with an excess risk of postoperative LCOS and severe arrhythmia [24]. Yau et al. found that during hypothermic CPB, the myocardium acquires energy through anaerobic metabolism, leading to the accumulation of lactic acid. After reperfusion, aerobic metabolism cannot be resumed immediately in cardiomyocytes owing to low temperature, but still anaerobic metabolism can occur for a short period. Whereas, patients with mild hypothermia CPB are less affected [25]. The study found that the inflammatory response mediated by CPB caused pulmonary vascular endothelial damage, which changed the pulmonary vascular reactivity. These cause excessive thromboxane production and reduced endogenous nitric oxide production, which can lead to pulmonary vasoconstriction and formation of pulmonary microthrombus. Further, these changes could induce pulmonary vascular resistance, which increases after CPB [26]. Then, the right ventricular afterload increases, which is significantly associated with right ventricular dysfunction and LCOS.

Obesity or high BMI is a common factor associated with poor prognosis. A propensity score-matched analysis found that obese (BMI, ≥30 kg/m^2^) patients who underwent surgery for type A acute aortic dissection had higher postoperative mortality rates. Moreover, a previous report stated that obesity was significantly associated with increased risk of LCOS and other postoperative morbidities [27]. However, in our study, there was no significant correlation between BMI and LCOS risk after cardiac surgery in CHD children. This correlation is different from the results of the abovementioned study on adults and we ascertain that the ‘obesity paradox’ may explain this inconsistency. The BMI of the CHD children in our study may be lower than that of the general population, and the patients with BMI > P95 percentile did not have such high obesity levels.

LCOS is associated with significantly high morbidity and mortality. In our study, the mortality rate (7.18% vs. 1.08%, *p* < 0.001), ICU stay > 3 days (89.24% vs. 33.35%, *p* < 0.001) and the duration of mechanical ventilator support > 48 h (93.21% vs. 44.5%, *p* < 0.001) were significantly higher in LCOS children compared to those without LCOS. A previous study showed that LCOS was associated with significantly high morbidity and mortality in adults [3].

Therefore, cardiac surgery can cause LCOS in children with CHD and result in poorer postsurgical outcomes. In the current study, we confirmed the impact of LCOS on postoperative clinical outcomes in CHD children.

## Limitations

This study had certain limitations. First, this is a retrospective study; the correlations may not confirm a causal relationship between the risk factors and LCOS. Second, we included patients from a single center only; hence, the application of these findings on other populations and institutions need to be reproduced in further large-scale multi-center studies. Since this is a single-center study, the impact of surgery outcomes and hospitalizations from other centers was not considered.

## Conclusion

In conclusion, our study provides clinically significant evidence to indicate a significant association of LCOS with postoperative clinical outcomes (including mortality, prolonged ICU stay, and extended mechanical ventilator support) in children with CHD. Circulation temperature, myocardial preservation using HTK, and usage of residual shunt after surgery were independent risk predictors for LCOS. Further multi-center studies toned to be conducted with a larger sample size to confirm our study results.

## Supplementary information

**Additional file 1: Table S1.** The Variance Inflation Factor of selected variables.

## Data Availability

The datasets generated during and/or analyzed during the current study are not publicly available due to the hospital regulation.

## Abbreviations

BMI: Body mass index
CHD: Congenital heart disease
CPB: Cardiopulmonary bypass
HTK: Histidine-tryptophan-ketoglutarate
ICU: Intensive care unit
LCOS: Low cardiac output syndrome
LVEF: Left ventricular ejection fraction
LVFS: Left ventricular fractional shortening
LVOTO: Left ventricular outflow tract obstruction
MR: Mitral regurgitation
RACHS-1: Risk Adjustment in Congenital Heart Surgery-1
RVOTO: Right ventricular outflow tract obstruction
TR: Tricuspid regurgitation
