# Supplementary material for: Risk factors for low cardiac output syndrome in children with congenital heart disease undergoing cardiac surgery: a retrospective cohort study
Source: BMC Pediatr. 2020 Feb 24;20:87. doi: 10.1186/s12887-020-1972-y (PMC7038550; doi:10.1186/s12887-020-1972-y)
Supplement: Supplementary file 1 — Additional file 1: Table S1. The Variance Inflation Factor of selected variables. [file 12887_2020_1972_MOESM1_ESM.docx]

Supplemental table 1. The Variance Inflation Factor of selected variables

| Variable | VIF |
| --- | --- |
| Aortic shunt：Left-to-right | 1.076 |
| Aortic shunt：Bi-directional | 1.100 |
| Atrial shunt：Left-to-right | 1.700 |
| Atrial shunt：Right-to-left | 1.391 |
| Atrial shunt：Bi-directional | 1.730 |
| Ventricular level shunt：Left-to-right | 1.666 |
| Ventricular level shunt：Right-to-left | 1.026 |
| Ventricular level shunt：Bi-directional | 1.831 |
| Age | 1.227 |
| TR | 1.150 |
| RACHS-1 risk grade | 1.201 |
| CPB duration | 1.634 |
| Residual shunt | 1.014 |
| LVOTO | 1.037 |
| RVOTO | 1.027 |
| Mitral insufficiency | 1.114 |
| Myocardial preservation | 1.270 |
| Circulating temperature：Mild hypothermia | 1.659 |
| Circulating temperature：Deep hypothermia | 1.168 |
| Circulating temperature：Middle and low temperature | 1.966 |
